# A Multivariate Linear Regression-Based Ultrasonic Non-Destructive Evaluating Method for Characterizing Weld Tensile Strength Properties

**DOI:** 10.3390/ma18091925

**Published:** 2025-04-24

**Authors:** Dazhao Chi, Ziming Wang, Haichun Liu

**Affiliations:** 1 State Key Laboratory of Precision Welding & Joining of Materials and Structures, Harbin Institute of Technology, Harbin 150001, China; 22s009128@stu.hit.edu.cn; 2 PipeChina Engineering Quality Supervision and Inspection Company, Beijing 100013, China; liuhc@pipechina.com.cn

**Keywords:** weld, tensile strength, ultrasonic testing, non-destructive testing

## Abstract

Destructive testing is a common method for obtaining tensile strength properties of welds. However, it is inconvenient to characterize the overall weld, and it cannot be applied to in-service structures. Non-destructive testing and evaluation (NDT&E) methods have the potential ability of overcoming these limitations. In this paper, an ultrasonic-based non-destructive evaluating method for weld tensile strength was proposed. Multiple sets of fully automatic welded X80 steel pipes were prepared. Acoustic signals from a total of 240 measurement points of the welds were collected, and ultrasonic characteristic parameters were subtracted through signal processing. Subsequently, tensile strength values were obtained through destructive testing. Using the ultrasonic and tensile test databases, a multivariate regression-based (MLR) non-destructive evaluation model was established to predict the tensile strength value. Based on this, in order to rapidly characterize the welds, a grading evaluation model was introduced. The grading evaluation result of the 240 measurement points indicates that the accuracy of the proposed method is 76.3%. In order to improve accuracy, a deep learning-based method could be used in the future.

## 1. Introduction

The mechanical properties of materials are the primary focus in engineering applications, especially in welding manufacturing [1,2,3]. To obtain these properties, such as tensile strength, impact toughness and hardness, the currently more reliable approach involves conducting destructive mechanical tests. However, destructive mechanical tests not only lead to a waste of raw materials but also make it impossible to obtain mechanical property indicators for structures in service. In response to this issue, researchers have been continuously attempting to obtain the mechanical properties of materials through non-destructive testing (NDT) methods in recent years.

During the research process of non-destructive characterization in material mechanics, the Magnetic Barkhausen Noise (MBN) technique has achieved certain results in hardness prediction [4,5] and thermal damage analysis [6,7]. Song [8] et al. designed a multivariate linear regression (MLR) model to predict the hardness of turbine gear carburized 18CrNiMo7-6. The result shows that several MBN signal features contribute to the calculation of hardness. Liu [9] et al. applied both MLR and BP neural network in the quantitative prediction of surface hardness of a ferromagnetic specimen of different temperatures. The result shows that the BP model has a smaller average prediction error than that of the MLR model. Zhang [10] et al. used MBN to determine the effect of welded rail properties and concluded that MBN signal has obvious advantages in characterizing the hardness distribution of the welded components. Avila [11] et al. used MBN to compare the microstructural, hardness and residual stress evolution of a two-pass friction stir welding (FSW) butt joint and found that MBN may be feasible to perform non-destructive quality evaluation. Apart from NDT methods based on magnetics, techniques based on eddy current have also received great attention [12,13]. Different from that of MBN, research based on eddy current has focused more on the characterization of the microstructural features of various materials. Saeed [14] et al. utilized scanning electron microscopic (SEM) observation, with X-ray diffraction (XRD) analysis as references, finding that the eddy current technique is very sensitive to the variety of phases that may result from different heat treatment conditions. Liu [15] et al. found that the volume fraction of strain-induced martensite and the hardness of cold rolled AISI 321 stainless steel can be estimated by the maximum amplitude and the maximum vertical component of eddy current. The high correlation coefficients of linear relationships between eddy current outputs and austenite fraction showed the ability to quantitatively characterize the microstructure.

Besides MBN and EC techniques, ultrasonic testing (UT) boasts rapid detection speed, high sensitivity, cost-effectiveness, versatility [16] in application and strong penetration capabilities, making it a highly effective non-destructive testing (NDT) technique for assessing material properties. Currently, among the ultrasonic methods used for characterizing the microstructure and mechanical properties of materials, laser ultrasonic testing (LUT) technology has been the most extensively studied [17,18,19,20], yielding several research achievements in aspects such as grain size [21,22,23], hardness [24,25] and heat treatment damage [26,27] of materials. Choi [28] et al. evaluated the abilities of ultrasonic velocity, ultrasonic attenuation coefficient and nonlinear parameters to characterize the grain size and the mechanical properties of 304L stainless steel. Apart from LUT, several studies based on conventional ultrasonic testing (UT) techniques have also been conducted. Zhu [29] et al. utilized a pulse-echo measurement system and concluded that the variation in ultrasonic attenuation is strongly associated with the microstructure evolution and the velocity ratio parameter has a linear relation with the average diameter of precipitates. Ma [30] et al. introduced a novel method for characterizing the thermal barrier coatings (TBCs) porosity through constructed BP-GPR algorithm. Several highly related characteristic parameters were extracted and the algorithm had high adaptability and accuracy to predict the TBCs porosity.

In summary, current research based on non-destructive methods to characterize the microstructure and mechanical properties of materials have primarily focused on grain size, hardness, thermal damage and residual stress, with the majority of studies centering on base materials. Also, the experimental results exhibit a relatively significant dispersion. However, there are limited studies on the characterization of the mechanical properties of the weld.

In this paper, multiple sets of multi-layer and multi-pass fully automatic welded seams were prepared. Acoustic signals of the weld structures were acquired at various measurement points using the ultrasonic pulse–echo method, and multiple ultrasonic characteristic parameters were obtained through signal processing. Subsequently, tensile specimens were prepared at each measurement point, and tensile strength values were obtained through destructive testing. Using data from the database, a multivariate linear regression (MLR) for tensile strength/ultrasonic characteristic parameters was established. Based on this, considering the actual weld failure situations, a grading inspection model for the tensile properties of welds was proposed to achieve ultrasonic non-destructive grading inspection of weld tensile strength.

## 2. Methodology

### 2.1. Principle

**Figure 1:**
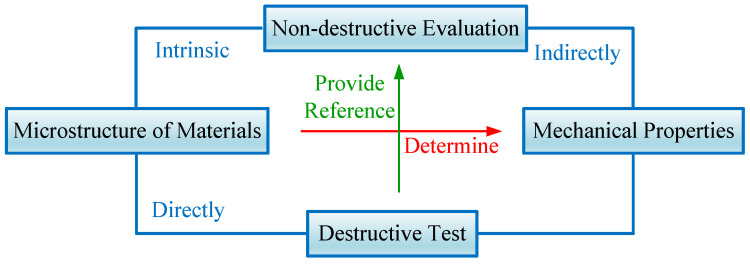
Intrinsic correlation between material microstructure, mechanical properties, destructive testing and non-destructive testing.

Figure 1 shows the intrinsic correlations between microstructure, mechanical properties and testing methods. The mechanical properties of materials are determined by their microstructures and can be directly obtained through destructive mechanical tests. Additionally, different microstructures of materials exhibit distinct acoustic properties. The research approach in this study is to establish a tensile strength/acoustic parameter regression model to achieve a non-destructive evaluation of material tensile strength, with reference to the results of destructive tensile tests. Subsequently, a tensile strength grading inspection model for weld seams is established to realize ultrasonic non-destructive grading inspection of weld seam tensile strength.

### 2.2. Research Process

**Figure 2:**
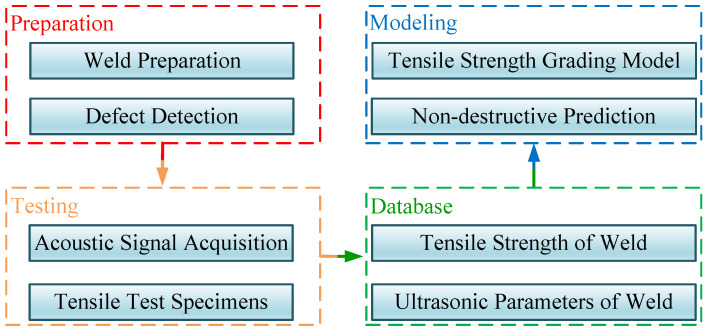
Experimental flowchart.

Figure 2 shows the work content of this study. It mainly comprises four parts: welding specimen preparation, welding specimen testing, database establishment and model establishment. In the welding specimen preparation section, welding seams of varying quality are first obtained by altering welding processes. Since this study does not involve the impact of macroscopic defects on weld seam tensile strength, ultrasonic non-destructive testing methods are employed post-welding to avoid defective locations. After obtaining the welding specimens, the ultrasonic signals at the measurement points are collected using the ultrasonic signal acquisition method designed in this study. Following this, tensile specimens are fabricated at the measurement points, and tensile strength values are obtained through destructive testing. Subsequently, the collected ultrasonic signals are analyzed to extract ultrasonic characteristic parameters, and a tensile strength database and an ultrasonic characteristic parameter database are established by integrating the tensile strength data from each measurement point. Finally, by analyzing and processing the database data, a tensile strength/acoustic characteristic parameter prediction model is established. Meanwhile, considering engineering application scenarios, this study proposes a grading inspection model for weld seam tensile strength properties and ultimately achieves weld seam tensile strength grading inspection.

## 3. Specimens and Test System

### 3.1. Preparation for Weld Specimen

**Figure 3:**
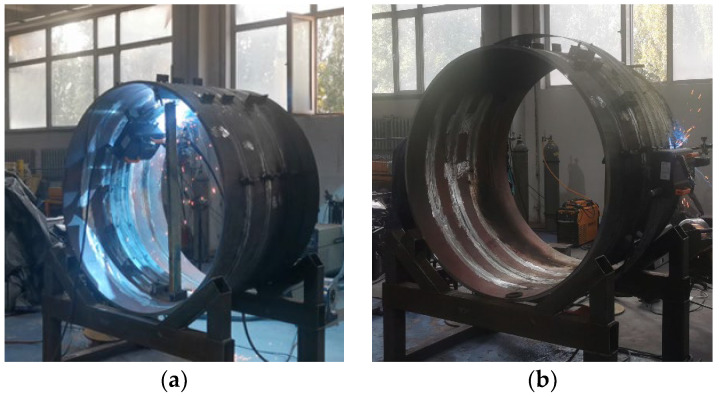
Preparation of welding parts and welding process. (**a**) Internal welding process (**b**) Multi-layer multi-pass weld.

The welding material used is steel piping with a diameter of 1219 mm. The welding method employed is a fully automatic multi-layer and multi-pass welding. The welding machine utilized is the Metal-Ant welding machine manufactured in China, with the welding power supply produced by Danish company Weld-Tech. Photographs of the welding process are shown in the figures, where Figure 3a depicts the internal welding process for the root pass and Figure 3b shows the external welding process.

After welding, the ultrasonic Time-of-Flight Diffraction (TOFD) method was used to detect defects in the weld seam. When significant continuous defects are present in the weld seam, the weldment is discarded. When dispersed defects are found in the weld seam, the locations of these defects are marked, and the weldment is then used for subsequent experiments and research.

### 3.2. Ultrasonic Test System

**Figure 4:**
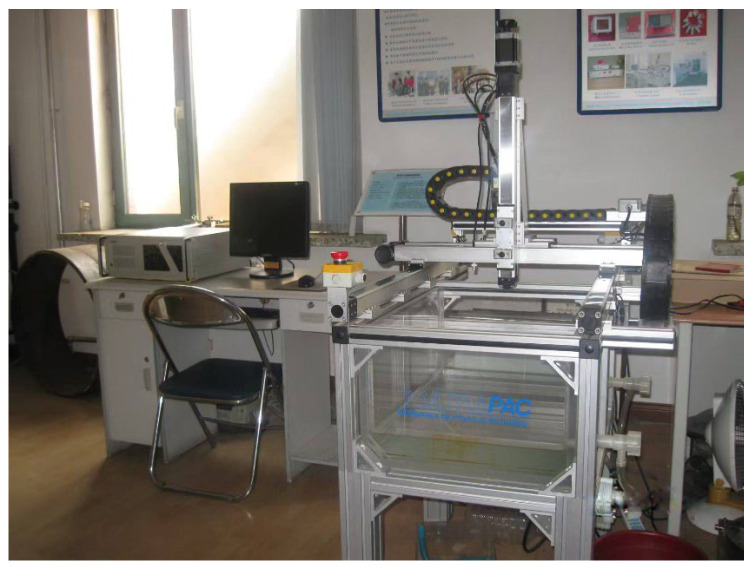
Ultrasonic test system.

An immersion ultrasonic testing system manufactured by Physical Acoustics in the United States was employed during the ultrasonic signal acquisition process, as shown in Figure 4. The thickness of the coupling layer was controlled with immersion, thereby enhancing the reliability of the test results.

### 3.3. Tensile Test System

**Figure 5:**
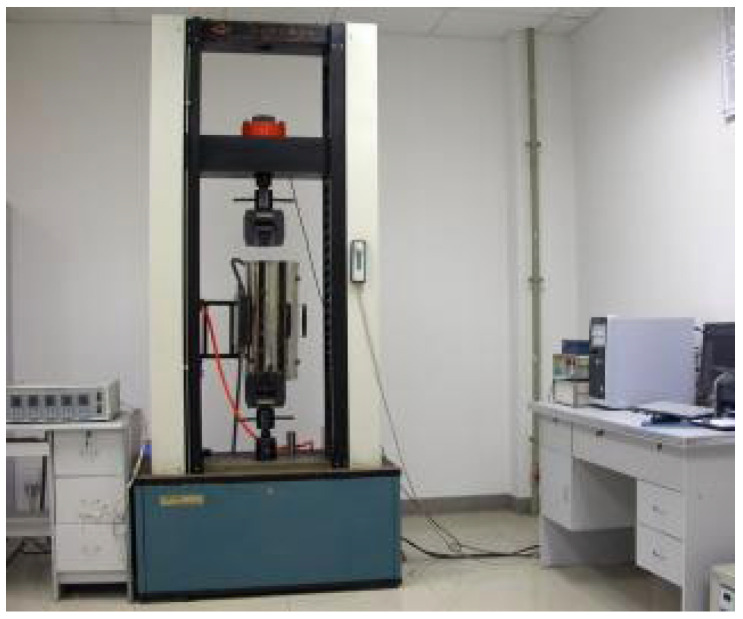
Tensile strength test system.

As shown in Figure 5, the tensile test was conducted using a CSS-44100 electronic universal testing machine manufactured in Sinotest Equipment Co. (Changchun, China) to obtain the tensile strength value of the specimen. The maximum load during the experiment was set at 100 kN.

## 4. Databases

In this study, the data primarily consist of two aspects: ultrasonic characteristic parameters database and tensile strength database, which are in a one-to-one correspondence according to the measurement points on the weld. This paper proposes a novel ultrasonic signal acquisition method and obtains various ultrasonic characteristic parameters through batch data processing. Based on the database, the ultrasonic non-destructive evaluation of weld tensile strength is conducted using mathematical statistics.

### 4.1. Ultrasonic Characteristic Parameters Database

#### 4.1.1. Method for Acquiring Ultrasonic Signals

**Figure 6:**
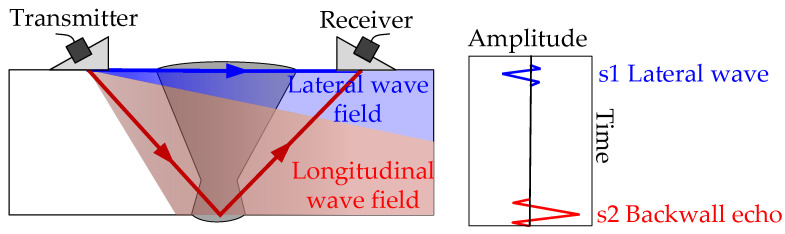
Illustration of ultrasonic signal acquisition and signal analysis.

As shown in Figure 6, this study employed a method of longitudinal wave oblique incidence with a fixed-distance dual-probe configuration, where one probe transmits and the other receives, to acquire ultrasonic signals in the weld seam. Based on the measured wedge sound velocity and material sound velocity, wedges with different angles for longitudinal waves were designed and fabricated according to Snell’s law. The ultrasonic signals acquired through this method contain two primary components: the lateral wave vibration signal (s1), which travels directly from the transmitting probe to the receiving probe, and the backwall echo vibration signal (s2). The lateral wave primarily reflects the interaction between the upper half of the weld seam’s structure and the ultrasonic waves, thereby establishing a connection with the structure and tensile strength of the upper half of the weld seam. The longer-propagation-time bottom-echo signal mainly reflects the interaction between the lower half of the weld seam’s structure and the ultrasonic waves, thus linking to the structure and tensile strength of the lower half of the weld seam.

**Figure 7:**
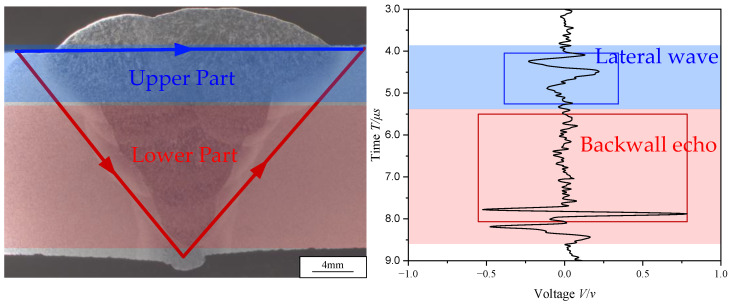
Weld cross-section and signal.

Figure 7 shows the cross-section of weld and signal of a measurement point. The entire weld is divided into two parts along the thickness direction: the upper part and the lower part. According to the propagation time of the ultrasonic waves, the influence of the microstructure in the upper part of the weld will be reflected in the blue region of the signal, while the influence of the microstructure in the lower part will be reflected in the red region. By extracting features from the ultrasonic vibration signals within different time windows, the microstructures of the upper and lower parts of the weld can be characterized.

#### 4.1.2. Ultrasonic Characteristic Parameters

Based on the analysis results of the ultrasonic signals obtained using the signal acquisition method employed in this study, four time–domain ultrasonic parameters were selected: the amplitude of the lateral wave (s1 Amp), the amplitude of the bottom echo wave (s2 Amp), the propagation time of the lateral wave (s1 Time) and the propagation time of the bottom echo wave (s2 Time). Among these, the amplitudes of the lateral wave and the bottom echo wave can reflect the energy loss when ultrasonic waves interact with the upper and lower sections of the weld joint; the propagation times of the lateral wave and the bottom echo wave can indicate the influence of the interaction between ultrasonic waves and the upper and lower sections of the weld joint on the propagation process of the ultrasonic waves. These ultrasonic characteristic parameters can all, to a certain extent, reflect the microscopic structure of the weld joint in different depth directions.

Batch processing on ultrasonic signals from 240 measurement points is performed. By applying time windows to temporally separate s1 and s2 in the time domain, the peak values and propagation times of signals were extracted.

### 4.2. Tensile Strength Database

In this study, a total of 240 measurement points were designated, and data were collected. Four tensile specimens were processed and fabricated at each measurement point, with two specimens taken from the upper half of the weld and the other two from the lower half. Tensile tests were conducted on the four tensile specimens from the same lateral point to obtain tensile property data. The data from the two groups, one from the upper half and the other from the lower half of the weld, were averaged separately to represent the tensile properties of the corresponding sections at the measurement point. A tensile strength database was then established.

### 4.3. Demonstration of Databases

**Figure 8:**
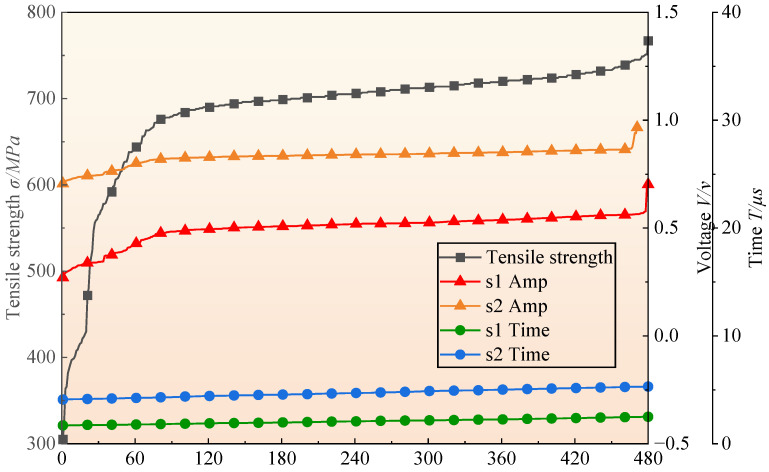
Database demonstration.

The total number of measurement points is 240. The number of tensile test specimens is 480 (upper and lower sections of the weld); therefore, the number of tensile strength values is 480. At each measurement point, two sets of ultrasonic signals are collected, resulting in 480 s1 Amp, s2 Amp, s1 Time and s2 Time values. All data were sorted in ascending order, as illustrated in Figure 8.

It should be noted that Figure 8 serves to visualize the range of data variation. Importantly, the tensile strength values in each column do not exhibit a one-to-one correspondence with the ultrasonic parameters. Thus, the trends shown in Figure 8 cannot be directly used to analyze the relationship between ultrasonic features and tensile strength properties.

## 5. Evaluation of Weld Tensile Strength

### 5.1. MLR Model

#### 5.1.1. Correlation Analysis and Variable Selection

**Figure 9:**
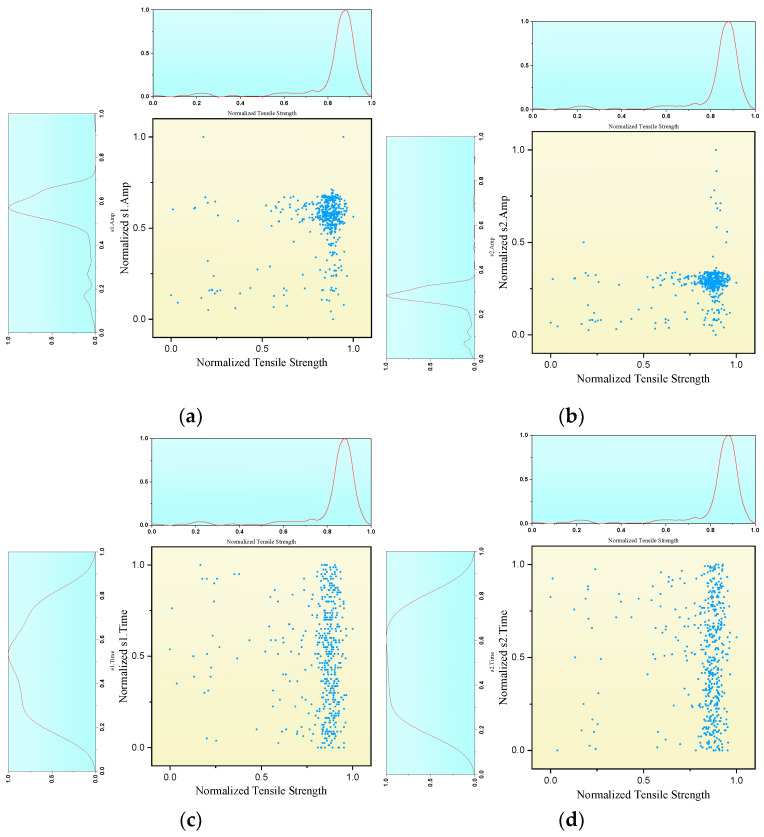
Correlation between tensile strength and different ultrasonic parameters. (**a**) s1 Amp (**b**) s2 Amp (**c**) s1 Time (**d**) s2 Time.

As shown in Figure 9, correlation analysis was conducted between tensile strength, lateral wave amplitude (s1 Amp), bottom echo amplitude (s2 Amp), lateral wave propagation time (s1 Time) and bottom echo propagation time (s2 Time). Scatter density plots illustrate the correlation between tensile strength and individual ultrasonic characteristic parameters. The spatial concentration of data points indicates the correlation, where tighter clustering corresponds to enhanced monotonic between variables. As Figure 9a,b demonstrates, data of s1 Amp and s2 Amp are relatively more correlated to that of tensile strength, for their distributions are more centered. In contrast, Figure 9c,d indicates that data of s1 Time and s2 Time are not correlated to that of tensile strength, for their distribution are random according to that of tensile strength.

Quantitatively calculated results are presented in Table 1. The correlation between direct wave propagation time and bottom echo propagation time with tensile strength data was extremely low, indicating that the velocity of sound is insensitive to changes in tensile properties. In contrast, the correlation between direct wave amplitude and bottom echo amplitude with tensile strength data was relatively high. As a result, s1 Amp and s2 Amp are selected for further analysis.

#### 5.1.2. MLR Prediction Model for Tensile Strength

**Figure 10:**
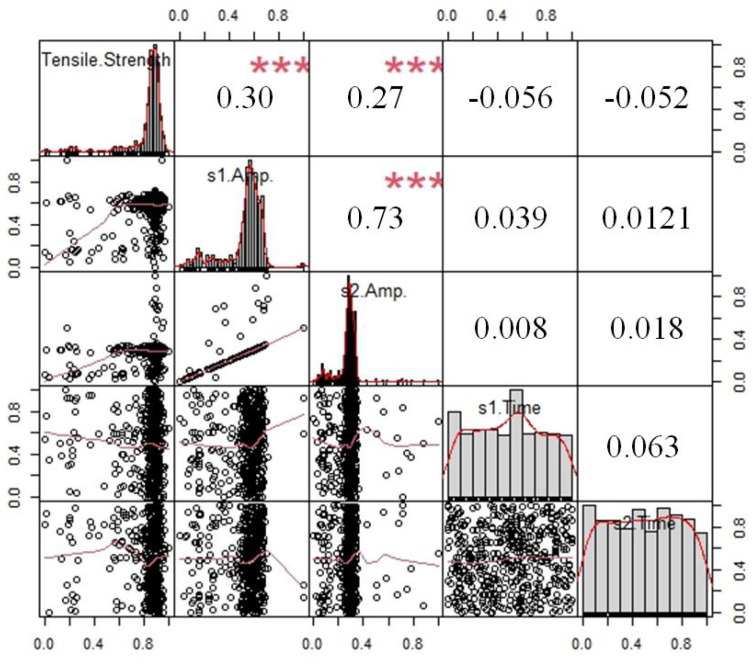
Multivariate regression results visualization (where symbol ‘***’ stands for 95% confidence interval).

Although the correlation between the lateral wave amplitude, the bottom echo amplitude and the tensile strength values is relatively high, it has not reached a significant level, suggesting that the error in directly establishing a multiple linear regression equation would be substantial. As shown in Figure 10, a set of regression analysis visualization graph shows that the residual of s1 Amp and s2 Amp are relatively high, with a strong regression coefficient of 0.30 and 0.27. In contrast, s1 Time and s2 Time contribute a low coefficient; the conclusion is the same as that of the correlation analysis. However, the multivariate linear regression result is not ideal, so further mathematical analysis is then employed.

To enhance the computational efficiency of the model and eliminate the scale influence among different parameters, normalization was applied to the tensile strength, s1 Amp and s2 Amp values. Subsequently, multiple linear regression analysis was conducted on the normalized data. The final regression model for weld tensile strength based on acoustic characteristic parameters is presented in Equation (1):

(1) $$Y=0.243X_{1}+0.164X_{2}+0.64738$$

where $X_{1}$ represents the value of normalized s1 Amp, $X_{2}$ represents the value of normalized s2 Amp.

### 5.2. Evaluation Model for Weld Tensile Strength Properties

**Figure 11:**
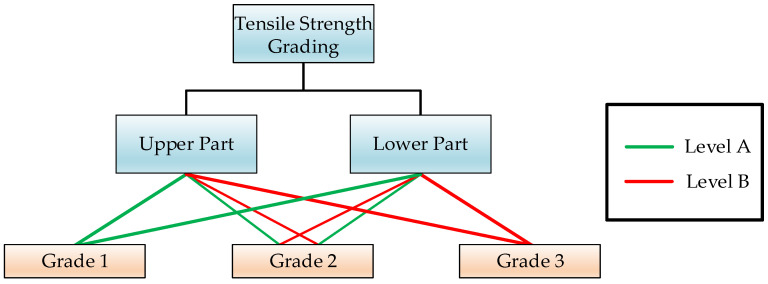
Grading evaluation model for tensile strength.

In this study, the tensile properties of the upper and lower halves of the weld seam were evaluated separately, and a classification method for assessing the tensile strength of the weld seam was proposed, as illustrated in Figure 11. Initially, a threshold tensile strength value for the weld seam, denoted as $\sigma_{0}$, was established. The tensile strength of the upper half of the weld seam was designated as $\sigma_{u}$, and that of the lower half was designated as $\sigma_{l}$. Subsequently, $\sigma_{u}$ and $\sigma_{l}$ were compared with $\sigma_{0}$. If both $\sigma_{u}$ and $\sigma_{l}$ exceeded the threshold $\sigma_{0}$, they were classified as Level A; conversely, if both were below $\sigma_{0}$, they were classified as Level B. At the same measurement point, there are four possible combinations of tensile properties for the upper and lower halves of the weld seam. When both the upper and lower halves exhibited Level A tensile strength properties, the comprehensive tensile strength performance at the measurement point was classified as Grade 1. When one half was Level A and the other was Level B, it was classified as Grade 2. When both halves were Level B, it was classified as Grade 3.

In this study, the threshold value $\sigma_{0}$ was selected to be 500 MPa. The accuracy of the grading evaluation model for weld tensile strength is presented in Table 2. As is shown in the table, destructive tensile test indicates that 206 measurement points were Grade 1 after grading evaluation. The non-destructive method calculated that 205 measurement points were Grade 1. A total of 160 measurement points exhibited consistent results. Using destructive testing results as the validation benchmark, the proposed method achieved correct identification in 160 out of 206 primary measurement points, with 46 points missed; thus, the accuracy is 77.7%. The accuracy of Grade 2 and Grade 3 were calculated following the same principle.

## 6. Discussion

In this section, an in-depth discussion on signal extraction, selection of ultrasonic characteristic parameters, grading evaluation model and the post development of the proposed method are presented

### 6.1. Signal Extraction

In this study, an oblique incidence and single-transmit-single-receive approach was employed to extract ultrasonic signals from the weld seam. The advantage of this signal acquisition method is that it allows for the acquisition of acoustic signals from both the upper and lower halves of the weld seam through one single signal acquisition. Analysis of the collected signal components revealed that the lateral wave signal primarily reflects the interaction between the ultrasonic wave and the microstructure of the upper half of the weld, while the bottom echo signal mainly reflects the interaction between the ultrasonic wave and the signal from the lower half of the weld.

### 6.2. Selection of Ultrasonic Characteristic Parameters

This study primarily selected two characteristic parameters: signal amplitude and propagation time. As is well known, signal amplitude primarily reflects the energy attenuation during the interaction between ultrasonic waves and the material microstructure. The more grain boundaries the ultrasonic wave traverses, the more pronounced the grain boundary reflection effect becomes, resulting in a lower signal amplitude. The propagation time of the signal mainly reflects the influence of the microstructure on the propagation process of ultrasonic waves, which is related to properties such as elasticity and density of the material.

Correlation analysis indicates that the correlation between signal amplitude and tensile strength is significantly greater than the correlation between propagation time and tensile strength. This may be due to the relatively small total distance traveled by the ultrasonic waves and insignificant differences in sound velocity.

### 6.3. Grading Evaluation Model

In engineering practice, there are primarily two modes of failure for weld structures: defects initiate in the upper half of the weld and gradually propagate to the lower half, or defects initiate in the lower half of the weld and continue to propagate within the lower half. Therefore, in structural inspection practices, it is necessary to pay separate attention to the microstructure and properties of both the upper and lower halves of the weld to maximize structural safety. Hence, this study proposes a classification method for assessing the tensile properties of welds.

The benefit of this approach is that it allows for a rapid judgment of whether the tensile strength properties at the inspection point meet the requirements during the inspection process. When the grading result is Grade 1, both the upper and lower halves of the weld meet the requirements, and no further inspection or analysis is needed. When the classification result is Grade 2, subsequent plans are designed based on the specific inspection results of the upper and lower halves of the weld. When the classification result is Grade 3, indicating that the tensile strength properties of the weld do not meet the requirements, measures such as repair welding or scrapping can be directly implemented to prevent structural safety accidents.

### 6.4. Post Development

Based on the distribution characteristics of the database and to eliminate the influence of scales and dimensions among different physical quantities, a normalized data processing approach was adopted for multivariate linear regression analysis. The advantage of this approach is that it can improve the accuracy of the tensile strength/acoustic characteristic parameter prediction model and significantly enhance the accuracy of classification detection. However, the improvement in accuracy achieved by this method is dependent on the distribution characteristics of the database. When the data distribution is discrete and the range is large, the effectiveness of the normalized data processing approach in improving the model’s accuracy will decrease.

The ultrasonic characteristic parameters employed in this study are conventional acoustic metrics. While these parameters exhibit principled correlation with material microstructural states, they may not represent the most optimal descriptors. Combination and feature engineering of ultrasonic parameters could potentially enhance the predictive accuracy. However, such multidimensional parameter optimization is most effectively implemented through deep learning. Therefore, our subsequent research will focus on developing deep learning-based methodologies to advance performance through automated feature abstraction and nonlinear relationship modeling.

## 7. Conclusions

1. In this study, the databases including ultrasonic characteristic parameters and tensile strength of 240 measurement points were established. Based on these databases, parameter selection based on correlation analysis and normalization was conducted to enhance the computational efficiency and eliminate the scale influence. Subsequently, a weld tensile strength prediction model was developed based on multivariate regression analysis.
2. In order to rapidly characterize the welds, a grading evaluation model was introduced. The test result of 240 measurement points indicates that the accuracy of the proposed method is 76.3%.
3. This study provides a comprehensive research framework for ultrasonic non-destructive characterization of weld mechanical properties. Apart from manual selection of ultrasonic parameters, deep learning-based methods will be researched in the near future.

## Figures and Tables

**Table 1:** Correlation analysis results.

		s1 Amp	s2 Amp	s1 Time	s2 Time
Tensile Strength	Coef.	0.29745	0.26545	−0.05644	−0.05244
	*p*-value	2.91182 × 10^−11^	3.48687 × 10^−9^	0.21711	0.25149

**Table 2:** Grading evaluation test results.

	Grade 1	Grade 2	Grade 3	Total
Tensile Test Results	206	24	10	240
Model Evaluated Results	205	22	13	240
Consistent Results	160	16	7	183
Accuracy	77.7%	66.6%	70.0%	76.3%

## Data Availability

The original contributions presented in the study are included in the article; further inquiries can be directed to the corresponding author.
